# Evaluation of the concentration of allergens from mites in fur of household dogs (Canis lupus familiaris) in Curitiba - PR - Brazil

**DOI:** 10.1186/1939-4551-8-S1-A164

**Published:** 2015-04-08

**Authors:** Dévaki De Assunção, Marconi Rodrigues De Farias, Rafael Rodrigues Ganho, Michelle Barbosa

**Affiliations:** 1 School of Agricultural Sciences and Veterinary Medicine, Pontifícia Universidade Católica Do Paraná, Brazil; 2 School of Medicine, University of São Paulo, Ribeirão Preto, Brazil

## Background

Allergens from house dust mites are perennial and also have enzymatic nature. They are commonly found in bedding, mattresses, pillows, bedroom floor and living room. In addition, they are often associated with sensitization and intensification in the symptoms of allergic rhinitis and asthma for susceptible individuals. Household dogs are continuously in contact with the ecological niches of house dust mites, and it has been observed that the microclimate between their skin and coat may favor its proliferation. Therefore, the present study aims to evaluate the concentrations of Der p 1, Der f 1 and Blo t 5, in the fur of household dogs, in order to check if they can serve as a reservoir of such allergens in the environment.

## Methods

Were selected 40 household dogs, regardless of breed, age and gender variables, which were bathed and brushed weekly, and also medicated regularly with acaricides and pulicides, besides living in the same niche as their owners. A sample of dust present in the fur of each dog was collected with a vacuum cleaner, passed across the length of their bodies, for two minutes. The samples were collected in separate filters transferred into plastic containers, sealed and kept frozen until analyzed by the ELISA method, using monoclonal anti Der p 1, anti Blo t 5 and anti Der f 1 (Indoor Biothecnologies-Chartottesville, USA). All the data were statistically analyzed, considering the minimum significance level of 5%.

## Results

Among the allergens studied, Der p 1 was the most commonly found (p <0.05). Of the 40 dogs evaluated 13 (32.5%) showed positive rates of Der p 1 on their fur and two (5%) were positive to Blo t 5. Average concentrations of Der p 1 and Blo t 5 were 0.14 ±0.73 μg.g^-1^ and 0.01±0.07 μg.g^-1^, respectively. No allergen Der f 1 was found in the fur of the studied dogs.

## Conclusions

The studied dogs can carry dust mites allergens in their fur, especially Der p 1, however in non sensitizing concentrations, which indicate that household dogs, kept under regular cleaning, are not a significant source of dust mites allergens.

